# The effects of apremilast on the QTc interval in healthy male volunteers: a formal, thorough QT study

**DOI:** 10.5414/CP202555

**Published:** 2016-06-10

**Authors:** Maria Palmisano, Anfan Wu, Mahmoud Assaf, Liangang Liu, C. Hyung Park, Ishani Savant, Yong Liu, Simon Zhou

**Affiliations:** 1 Celgene Corporation, Summit, NJ, USA,; 2 Novartis Institutes for BioMedical Research, Shanghai, China, and; 3 Bristol-Myers Squibb, Princeton, NJ, USA

**Keywords:** apremilast, inflammatory autoimmune disorders ‒ phosphodiesterase 4 inhibitor ‒ QTc interval

## Abstract

Objective: This study was conducted to evaluate the effect of apremilast and its major metabolites on the placebo-corrected change-from-baseline QTc interval of an electrocardiogram (ECG). Materials and methods: Healthy male subjects received each of 4 treatments in a randomized, crossover manner. In the 2 active treatment periods, apremilast 30 mg (therapeutic exposure) or 50 mg (supratherapeutic exposure) was administered twice daily for 9 doses. A placebo control was used to ensure double-blind treatment of apremilast, and an open-label, single dose of moxifloxacin 400 mg was administered as a positive control. ECGs were measured using 24-hour digital Holter monitoring. Results: The two-sided 98% confidence intervals (CIs) for ΔΔQTcI of moxifloxacin completely exceeded 5 ms 2 – 4 hours postdose. For both apremilast dose studies, the least-squares mean ΔΔQTcI was < 1 ms at all time points, and the upper limit of two-sided 90% CIs was < 10 ms. There were no QT/QTc values > 480 ms or a change from baseline > 60 ms. Exploratory evaluation of pharmacokinetic/pharmacodynamic data showed no trend between the changes in QT/QTc interval and the concentration of apremilast or its major metabolites M12 and M14. Conclusions: Apremilast did not prolong the QT interval and appears to be safe and well tolerated up to doses of 50 mg twice daily.

## Introduction 

Apremilast is an orally available compound approved for the treatment of active psoriatic arthritis (PsA) and moderate-to-severe plaque psoriasis. Apremilast inhibits the phosphodiesterase 4 (PDE4) enzyme; PDE4 is the predominant cyclic adenosine monophosphate phosphodiesterase (cAMP) in inflammatory cells. This inhibition of PDE4 causes a rise in intracellular cAMP levels and modulates expression of a wide array of inflammatory mediators [1, 2, 3, 4]. Recent clinical studies have shown that apremilast can be used in the treatment of PsA and psoriasis and has acceptable tolerability and safety profiles [5, 6, 7, 8, 9, 10]. 

Apremilast is extensively metabolized and excreted in the urine and feces, predominantly as metabolites M12, M14, and M16. The major circulating metabolite, M12 (*O*-des-methyl-[S]-CC-10004-glucuronide) is eliminated with a longer half-life of ~ 16 hours than the parent compound, which has a half-life of ~ 6 – 9 hours. The metabolite to total radioactivity ratio in humans was ~ 39% for M12 and < 10% for M14 and M16 [11]. All three are glucuronide metabolites and are not pharmacologically active [11]. 

Preclinical in-vivo studies in dogs and in-vitro studies in human embryonic kidney (HEK 293) cells expressing the human ether-a-go-go related gene (hERG) showed that apremilast had a substantial safety margin in terms of risk of cardiac arrhythmias. In-vitro studies in HEK293 cells showed that apremilast inhibited the hERG current with an estimated half maximal inhibitory concentration (IC_50_) of 184.2 µM (84.8 µg/mL) and Hill coefficient of 0.9. This IC_50_ value is > 2 orders of magnitude greater than the maximum plasma concentration (C_max_) values observed in healthy male subjects and in PsA or rheumatoid patients at the highest doses studied [5, 6, 7, 8]. Prolongation of the QT interval is associated with a rare but dangerous ventricular arrhythmia, torsade de pointes, and drug-induced prolongation of the QT interval represents a significant risk. The current study was undertaken to provide definitive evaluation of the clinical potential for prolongation of the QT interval with apremilast [12]. 

The main objective of this study was to evaluate the effect of oral administration of apremilast on the placebo-corrected change-from-baseline QT interval using an individual correction method (ΔΔQTcI). In addition, the study evaluated the ΔΔQT interval using the Fridericia’s correction method (ΔΔQTcF) and the Bazett’s correction method (ΔΔQTcB) as well as heart rate (HR), PR interval, QRS interval, uncorrected QT interval (QT), and morphological patterns following apremilast treatment. Moxifloxacin was used as the positive control to ensure that the study has the ability to detect whether apremilast has an effect on the QTc interval. An exploratory analysis was conducted to evaluate the pharmacokinetic/pharmacodynamic (PK/PD) relationship between the PK exposure of apremilast and its metabolites (M12, M14, and M16) and the QTc changes. 

## Methods 

### Study design 

This was a single-center, randomized, crossover study with 4 treatments, 4 periods, and 4 sequences conducted in healthy male subjects. Apremilast (Otezla; Celgene Corporation, Summit, NJ, USA) was administered in a double-blind manner with matching placebo, and moxifloxacin was administered in an open-label manner. The core central electrocardiogram (ECG) laboratory staff, including those reading the ECGs, was blinded to treatment, sequence time, and subject identifiers. Data management, analysis, and clinical portions of the study were conducted by Quintiles (Overland Park, KS, USA); ECGs were centrally read by Quintiles ECG Services (Mumbai, India). 

Subjects were randomized to 1 of 4 treatment sequences. Eligible subjects reported to the clinic on day –2 and remained in the clinic until day 6 for each of the 4 treatment periods. Subjects were housed at Quintiles Phase I Services (Overland Park, KS, USA). Each treatment period had its own assessment of baseline ECGs, which were collected on day –1. There was a minimum of a 7-day washout (time from last dose to next dose) between the treatment periods. An end-of-study visit was conducted 7 (± 1) days after the last day of the last treatment period (day 6 of period 4). 

The 4 treatments evaluated in this study included an apremilast-matched placebo administered twice daily (b.i.d.) for 9 doses (~ 5 days), a clinically relevant dose of apremilast 30 mg administered b.i.d. for 9 doses, a supratherapeutic dose of apremilast 50 mg administered b.i.d. for 9 doses, and placebo administered on days 1 – 4 followed by a single dose of moxifloxacin 400 mg on day 5. Subjects fasted for at least 8 hours before the morning dose or before the start of Holter monitoring on days –1 and 5. 

### Subjects 

Male subjects in good health, as determined by medical history, physical examination, vital signs, and laboratory tests at screening, were eligible to participate in the study. In addition, subjects had to have a 12-lead ECG, including a Fridericia’s correction (QTcF) or Bazett’s correction (QTcB) value ≤ 430 ms, and a body mass index between 19 and 30 kg/m^2^ (inclusive) and body weight between 50 and 115 kg. Subjects were not eligible if they had a first-degree relative with long QT syndrome, abnormal blood pressure for their age, abnormal ECG findings or unfavorable or consistently accurate QT measurements, or a neuromuscular artifact that could not be readily eliminated. Subjects were also excluded for having a history of cardiovascular disease, autonomic dysfunction, acute or chronic bronchospastic disease, or clinically significant drug allergy or atopic allergy. 

The study was powered for the primary endpoint of the time-matched placebo-corrected change from baseline in QTcI. A sample size of 49 subjects with triplicate ECGs per time point provided at least 80% power. 

This study was conducted in accordance with the Declaration of Helsinki Good Clinical Practice: Consolidated Guideline approved by the International Conference on Harmonisation (ICH), as required by and described in Title 21 of the Code of Federal Regulations (CFR) parts 50, 54, 56, 312 subpart D, and 314. An independent institutional review board (Mid*Lands Institutional Review Board, Leawood, KS, USA) reviewed and approved the clinical study protocol, informed consent document(s), and other relevant study documents. 

### Pharmacokinetic analysis 

PK parameters were derived using noncompartmental methods with WinNonlin^®^ Professional version 5.1.2 (Pharsight Corporation, Mountain View, CA, USA). Serial blood samples for plasma PK analysis were collected on day 5 of each treatment period at predose (0 hour) and 0.5, 1, 1.5, 2, 3, 4, 6, 12, and 23 hours following the morning dose. For determination of PK parameters (terminal elimination rate constant (K_el_), time to maximum plasma concentration (t_max_), and terminal half-life (t_1/2_)), plasma concentrations for apremilast and its major metabolites below the limit of quantitation (BLQ) were treated as missing except for the calculation of area under the plasma concentration-time curve (AUC). To calculate AUC, BLQ or missing concentrations in the beginning and end of profile were replaced with zero, and BLQ or missing concentrations in the middle of the profile were interpolated. 

### Analytical methods 

Blood samples were collected in lithium heparin tubes and plasma was separated. The plasma samples, fortified with Sorensen’s buffer (25 mM citrate, pH 1.5) containing amastatin 20 µM (1 : 1/v : v), were analyzed by two validated liquid chromatography/tandem mass spectrometry methods, one for determination of apremilast and the other for its metabolites M12 (CC-16166), M14 (CC-16793), and M16 (CC-16557). 

### Pharmacodynamic analysis 

Digital Holter monitors were used to record high-resolution (1,000 Hz) 12-lead ECGs for ~ 24 hours continuously from 30 minutes predose to 23 hours following the morning dose on day –1 and day 5 of each treatment period. Triplicate ECGs were extracted at 2-minute intervals from the Holter data on day –1 of period 1 at matched time points as for day 5; on day 1 of each period at 1, 0.5, and 0 hours prior to the first dose; and on day 5 of each period at predose and at 0.5, 1, 1.5, 2, 3, 4, 6, 12, and 23 hours following the morning dose. 

ECGs were analyzed for ventricular HR, PR, QRS, RR, and QT intervals and morphological changes. Mean of the triplicate ECGs at each nominal time point was used for all reporting and analysis; however, individual values were used to calculate the correction coefficient (γ) for QTcI. The QTc corrections were individual correction by QTcI = QT/RR^γ^, Fridericia’s by QTcF = QT/RR^0.33^, and Bazett’s by QTcB = QT/RR^0.5^. Individual correction coefficient, γ, was estimated using linear regression of log QT on log RR for each subject using all baseline ECG assessments collected before day 1 dosing in period 1 within a subject. Change from baseline was calculated as the average of the three predose assessments on day 1 of the corresponding treatment period subtracted from the value (i.e., QTc) on day 5 at a given time t. Double-delta (ΔΔ) values for various cardiac parameters (i.e., ΔΔQTcI, ΔΔQTcB, etc.) were determined as the change from baseline value relative to placebo at the same scheduled time. 

### Central tendency analysis for QT/QTc 

Analysis of ECG data was carried out in accordance with the ICH E14 guidance [12]. The primary analysis assessed the effect of apremilast 30 mg b.i.d. and 50 mg b.i.d. on cardiac repolarization, as measured by the time point with the largest upper one-sided 95% confidence limit for the ΔΔQTcI using a linear mixed model with fixed effects for treatment, period, sequence, scheduled time, and baseline value as a covariate, and interaction of treatment by scheduled time with scheduled time as a repeated measure. At each time point, difference in least-squares (LS) means was estimated between the LS means of each apremilast dose and placebo (with placebo as reference). Two-sided 90% confidence intervals (CIs) were produced for the comparisons. 

The effect of QTcI for moxifloxacin was assessed at 1, 1.5, 2, 3, and 4 hours postdose to assess assay sensitivity. At each time point, the ΔΔ value was estimated between the LS means of moxifloxacin and placebo. Two-sided 98% CIs were produced for the comparisons. If at least one of the CIs completely exceeded 5 ms, then the assay was declared sensitive. 

For secondary analyses, a Student’s t-test was performed comparing each dose of apremilast vs. placebo and between moxifloxacin and placebo at each time point. Two-sided 90% CIs were produced for the comparisons. The effect of apremilast on QTcF, QTcB, HR, PR, and QT was assessed using the same linear mixed model from the primary analysis. For the time-averaged analysis, the mixed analysis of covariance (ANCOVA) model was used to compare the overall change from baseline across all time points between the placebo and two active treatment groups. The model included treatment group, period, sequence, and baseline ECG interval as a covariate, and subject nested within sequence as a random effect. The two-sided 90% CIs based on the mixed ANCOVA model were constructed for the pair wise mean differences between each active treatment group and placebo using the residual error of the mixed ANCOVA model. 

### Categorical analysis 

Clinically noteworthy ECG events were categorized by (a) absolute values for QT, QTcI, QTcB, and QTcF on day 5 into groups < 450 ms, 450 – 480 ms, > 480 – 500 ms, and > 500 ms; (b) the maximum positive change from baseline to day 5 in QT, QTcI, QTcB, and QTcF into groups with increases ≤ 0 ms, 1 – 30 ms, > 30 – 60 ms, and > 60 ms; (c) any HR value > 120 bpm or < 60 bpm; (d) any increase from baseline in PR > 25% when PR is > 200 ms; (e) any increase from baseline in QRS > 25% when QRS is > 100 ms; (f) any decrease from baseline in HR > 25% when HR is < 50 bpm; and (g) any increase from baseline in HR > 25% when HR is > 100 bpm. If a subject experienced > 1 episode of a particular outlier event, the subject was counted only once for that event. 

The morphological waveform analysis of the Holter data was made programmatically at each time point for new onset cardiac abnormalities, defined as not present on any baseline ECG (day –1) but present on any on-treatment ECG (day 5). Abnormalities were summarized using the standardized terms defined by the central cardiac laboratory and also broadly classified as morphological, conduction, and rhythm abnormalities. 

### Exploratory PK/PD relationships 

The PK/PD analysis was performed using a linear mixed-effect model to explore the relationship between the ΔΔQTcI and plasma concentrations for apremilast, M12, M14, and M16, and to estimate the population slope (β) and standard error of slope (SE_β_). The expected maximum ΔΔQTcI effect for each dose and each analyte was estimated using the slope and average C_max_ with one-sided upper limit 95% CIs. 

Statistical analyses were performed with SAS^®^ version 9.1.3 (SAS Institute, Inc., Cary, NC, USA). Graphics were prepared with SAS version 9.1.3 or SigmaPlot^®^ 9.0 (Systat Software, Inc., Point Richmond, CA, USA). 

## Safety 

Safety assessments were performed at clinic check-in, periodically throughout each treatment period, and at the end-of-study visit and included the measurement of vital signs, recording of standard 12-lead ECGs, collection of samples for clinical laboratory tests, and physical examinations. Adverse events (AEs) and concomitant medication use were assessed and recorded throughout the study. 

## Results 

### Subject disposition 

The 60 healthy adult male subjects enrolled in this study had a mean (SD) age of 29.0 (7.95) years (range 19 – 47 years); a mean (SD) weight of 83.21 (10.39) kg (range 61.7 – 103.3 kg); a mean (SD) height of 179.53 (6.84) cm (range 166.0 – 197.0 cm); and a mean (SD) body mass index of 25.81 (2.84) kg/m^2^ (range 20.0 – 30.3 kg/m^2^). 39 (65.0%) subjects were white, 20 (33.3%) were black, and 1 (1.7%) was American Indian/Alaskan Native. 

All 60 subjects received ≥ 1 dose of study drug. Of these, 52 (86.7%) completed all study procedures and received all planned doses of study drug. Eight (13.3%) subjects prematurely withdrew from the study: 5 (8.3%) withdrew consent, 2 (3.3%) were withdrawn due to AEs, and 1 (1.7%) was lost to follow-up. 

### PK results 

**Figure 1.**
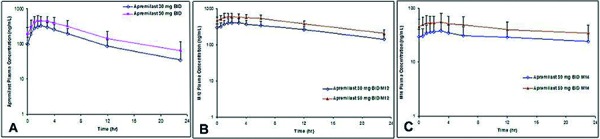
Mean (+ SD) plasma concentration of (A) apremilast, (B) M12, and (C) M14 by time and treatment on day 5 (semi-logarithmic scale). b.i.d. = twice daily.

The mean plasma concentration-time profiles of apremilast, M12, and M14 are illustrated in Figure 1. M16 concentrations were either very low or undetectable in all subjects; therefore, no PK analysis was performed. The PK parameters for apremilast, M12, and M14 are included in Table 1. Apremilast appeared to decline in a monophasic manner, and the mean t_1/2_ appeared to be similar (6 – 7 hours) across both doses. Consistent with prior studies, M12 is the most abundant circulating plasma metabolite of parent apremilast, with AUC and C_max_ being ~ 1.7-fold and ~ 1.25-fold, respectively, of those values for apremilast. Metabolite M14 is present at a much lower concentration than apremilast, with AUC and C_max_ having a metabolite to parent ratio that is ~ 0.16 and ~ 0.12, respectively, for both doses studied. 

### PD results 

**Figure 2.**
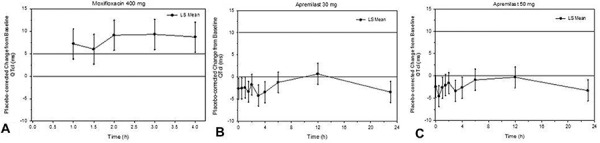
Change from baseline and placebo-corrected QTcI vs. time: (A) moxifloxacin 400 mg mean two-sided 98% CI and apremilast (B) 30 mg b.i.d. and (C) 50 mg b.i.d. mean two-sided 90% CI. CI = confidence interval; QTcI = QT interval using an individual correction method.

The median individual correction factor (γ) for QTcI was 0.2578, which was lower than the commonly used population correction factors, namely Fridericia’s (0.33) and Bazett’s (0.5). Figure 2 presents the time-matched, placebo-corrected change-from-baseline QTcI (ΔΔQTcI) for moxifloxacin and apremilast. Mixed-effect model analysis showed that LS mean ΔΔQTcI values for moxifloxacin were > 5 ms at all time points, ranging from 6.0 to 9.3 ms, between 1 and 4 hours postdose. The lower limit of the associated two-sided 98% CIs ranged from 2.7 to 5.9 ms and was > 5 ms at each time point from 2 – 4 hours postdose. This establishes the assay sensitivity required for a thorough QTc study. 

All LS mean ΔΔQTcI values for apremilast were < 1 ms, ranging from –4.2 to 0.7 ms for the 30 mg dose and –4.6 to –0.3 ms for the 50 mg dose. The upper limit of the two-sided 90% CI for both doses ranged from –2.2 to 3.1 ms, staying well below 10 ms at all time points. 

Similarly, LS mean values of time-matched, placebo-corrected change-from-baseline QTcF and QTcB (ΔΔQTcF and ΔΔQTcB) for moxifloxacin were > 5 ms at all time points between 1 and 4 hours postdose. The lower limits of the two-sided 98% CIs of LS mean ΔΔQTcF and ΔΔQTcB were > 5 ms from 2 – 4 hours postdose. For both apremilast doses, all LS mean ΔΔQTcF values were < 1 ms and all LS mean ΔΔQTcB values were < 5 ms. The upper limit of the two-sided 90% CI for both corrections were well below 10 ms at all time points. The results obtained with a model-independent Student’s paired t-test and comparisons of time-averaged ΔΔQTcI, ΔΔQTcF, and ΔΔQTcB were consistent with the results from the linear mixed-effect model on QTc prolongation. 

Categorical analysis of QTc data show that no subjects in any treatment group had an absolute value in QTcI, QTcF, or QTcB > 480 ms or change from baseline value > 60 ms. The waveform analysis of the thorough QT data also shows that the overall number of cardiac abnormalities was low, with only sinus tachycardia and right axis deviation occurring in > 1 subject. These events were isolated and not considered clinically significant. 

### PK/PD relationship 

**Figure 3.**
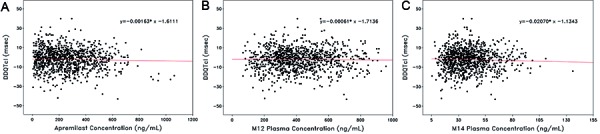
Pooled change from baseline and placebo-corrected QTcI interval vs. (A) apremilast, (B) M12, and (C) M14 concentrations. QTcI = QT interval using an individual correction method.

The relationship between ΔΔQTcI and plasma concentrations of apremilast, M12, and M14 for subjects in the PK and evaluable populations are illustrated in Figure 3. Visually, there was no apparent relationship between ΔΔQTcI and plasma concentrations. Linear regression lines and equations based on a random effect model are shown. The estimated slopes (SE) are –0.0016 (0.0027) for apremilast, –0.0006 (0.0028) for M12, and –0.0207 (0.0343) for M14 concentrations. The figures demonstrate no linear relationship between ΔΔQTcI and plasma concentrations of apremilast or the metabolites; thus, QTc intervals did not increase when apremilast plasma concentrations increased. 

### HRs and PR intervals 

Although steady-state apremilast did not significantly increase QT/QTc intervals at doses up to 50 mg b.i.d., there were small changes in HRs and PR intervals for both apremilast treatment groups. Time-matched, baseline-adjusted LS mean HR (ΔΔHR) increased 3.1 bpm at 2 hours after administration of apremilast 30 mg dose and 4.4 bpm at 3 hours following administration of the 50 mg dose. Time-matched, baseline-adjusted LS mean PR interval (ΔΔPR) decreased 2.8 and 3.5 ms at 2 hours postdose for both apremilast doses. Furthermore, scatter plots of ΔΔHR and ΔΔPR interval with increasing plasma concentration of apremilast did not show any apparent relationship. Overall, the increases in HR and decreases in PR interval were minimal, thus indicating that these changes are not considered clinically meaningful. 

### Safety evaluations 

A mild AE is defined as an event in which intervention is not indicated, activities of daily living are minimally or not affected, and no or minimal intervention/therapy may be required. A moderate AE is defined as an event in which local or noninvasive intervention is not indicated, there is more than minimal interference with activities of daily living but the patient is able to carry out daily social and functional activities, and drug therapy may be required. No deaths or serious AEs were reported during the study. Two (3.3%) subjects were withdrawn due to AEs of increased alanine aminotransferase (moderate and no suspected relationship to apremilast treatment) and nausea (moderate and suspected relationship to study treatment). Overall, 283 AEs were reported in 57 (95.0%) subjects during the study. The most frequently reported treatment-related AE was headache, which occurred in 39 (65.0%) subjects overall. There were no reports of cardiac-related treatment-emergent AEs. Most of the treatment-emergent AEs were mild (33/60 subjects) or moderate (23/60 subjects). Overall, no trends or clinically meaningful changes were observed in clinical laboratory tests, vital signs, or ECG intervals throughout the study. 

## Discussion 

This thorough QT/QTc study was performed in compliance with the principles of the ICH E14 guidance and was designed to adequately assess any effect on QTc duration following multiple administration of apremilast at doses of 30 mg b.i.d. or 50 mg b.i.d. in healthy male subjects. The analyses showed that apremilast administered in multiple doses at 30 mg or 50 mg had little or no effect on QT/QTc interval. The primary analysis of central tendency showed that all placebo-corrected, change-from-baseline mean QTcI values were < 1 ms, with the upper limit of all two-sided 90% CIs staying well below 10 ms. The secondary analysis, based on QTcF and QTcB, provided similar results. In addition, time-matched, baseline-adjusted HRs and PR intervals showed neither a clinically significant change compared with placebo nor a relationship to the apremilast plasma concentration. Consistent with these findings, the results of categorical analyses indicate that apremilast in this dose range does not prolong the QT interval. No subject had a maximum on-treatment QT/QTc interval > 480 ms or a change from baseline > 60 ms for any QT/QTc evaluation while receiving apremilast. 

The apremilast doses studied represent the therapeutic dose for the indications of PsA and psoriasis (i.e., 30 mg b.i.d.) and a supratherapeutic dose (i.e., 50 mg b.i.d.). The study was powered for primary analysis of the time-matched, placebo-corrected change from baseline in QT interval following individual correction for HR (QTcI). All 53 subjects met the requirements for inclusion in the analysis, including 52 subjects who completed all study procedures and received all study drugs plus 1 subject who completed the placebo and apremilast 30 mg b.i.d. treatment phase, which exceeded the requirement of the study for adequate power. 

The lack of a relationship between plasma concentrations of apremilast and its major metabolites, M12 and M14, and the changes in QTcI intervals in the PK/PD analysis also demonstrate that the QT/QTc interval was unaffected by increasing plasma concentrations of apremilast or its metabolites. The linear regression analysis of the PK concentrations in relation to ΔΔQTcI resulted in negative slope values for each of the 3 PK analytes studied (i.e., apremilast, M12, and M14), showing a slight decrease in ΔΔQTcI as the concentrations increased (Figure 3), which is not of clinical concern. Maximum ΔΔQTcI expected at the average C_max_ (for each of the 3 PK analytes), based on the PK/PD model, were all negative values ranging from –0.28 to –1.3 and the upper limit of the 95% CIs were all < 3 ms. Apparent lack of a direct PK/PD relationship provides strong evidence that multiple administration of apremilast in this dose range does not prolong the QT interval. 

Consistent with findings in healthy subjects, a voltage-clamp study in HEK-293 cells (which lack endogenous I_Kr_) that stably express hERG potassium channel demonstrated that apremilast has low affinity for the hERG channel. Furthermore, a structure-activity relationship study has shown that the structure of a PDE4 inhibitor can be optimized for high-potency PDE4 inhibition and minimize or eliminate its affinity for hERG channel by targeted modification of pharmacophores [13]. The structure of apremilast was optimized for PDE4 inhibition and minimal affinity for the hERG channel [14]. 

PK data provided evidence that the exposure of apremilast had achieved the level that is appropriate for a thorough QT/QTc evaluation. The t_max_ and t_1/2_ values for apremilast and its metabolites were consistent with those reported previously, and the exposure increased proportionally with increasing doses of apremilast (~ 1.4 times for C_max_; ~ 1.5 times for AUC) [5, 6]. The dose-dependent increase of exposures did not lead to prolongation of QTc interval or cardiac AEs in healthy male subjects. In the current study, mean apremilast exposures in healthy subjects administered apremilast at a supratherapeutic dose of 50 mg b.i.d. (geometric mean C_max_ = 507.2 ng/mL; geometric mean AUC_0–12_ = 3,451 ng×h/mL; n = 55) were comparable to the exposures observed in patients with rheumatoid arthritis or PsA administered apremilast 30 mg b.i.d. (geometric mean C_max_ = 554 ng/mL; mean AUC_0–12_ = 3,670 ng×h/mL; n = 15), which is the therapeutic dose for patients with PsA and/or psoriasis [15]. Of note, the maximum apremilast exposures in healthy subjects administered 50 mg b.i.d. in the current study (maximum C_max_ = 1,062 ng/mL; maximum AUC_0–12_ = 9,350 ng×h/mL) exceeded the maximum apremilast exposures reported in PsA and psoriasis patients administered 30 mg b.i.d. (maximum C_max_ = 981 ng/mL; maximum AUC_0–12_ = 8,280 ng×h/mL) [15]. Therefore, apremilast does not lead to QTc prolongation at the therapeutic dose (30 mg b.i.d.) and will unlikely lead to prolongation of QTc at doses beyond those evaluated. 

Although this thorough QT/QTc study was performed in healthy male subjects following multiple administration of apremilast at doses of 30 mg b.i.d. and 50 mg b.i.d., the negative effect on QTc can be extrapolated to all populations such as females and patients. As discussed above, apremilast exposure in healthy subjects administered 50 mg b.i.d. in the current study exceeded that in PsA and psoriasis patients (both males and females) administered 30 mg b.i.d.. This conclusion is further supported by lack of clinically significant ECG abnormalities in large-scale clinical trials. 

In summary, this study demonstrates that apremilast does not increase the QTc interval at therapeutic and supratherapeutic doses, based on the criteria of ICH E14 guidance. No trend or relationship was observed between the changes in QT/QTc interval and the concentrations of apremilast or its major metabolites M12 and M14. In contrast, moxifloxacin significantly increased the QT/QTc interval, confirming its proarrhythmic activity and sensitivity of the study. Therefore, apremilast does not lead to QTc prolongation at the therapeutic dose (30 mg b.i.d.) and will unlikely lead to prolongation of QTc at doses beyond the therapeutic dose. 

## Acknowledgments 

The authors thank their colleague Xiaomin Wang, PhD, for technical contributions to the bioanalysis. The authors received editorial support in the preparation of this manuscript from Prachi Wickremasingha, PharmD, funded by Celgene Corporation. The authors, however, directed and are fully responsible for all content and editorial decisions for this manuscript. 

## Source of funding 

This study was sponsored by Celgene Corporation. 

## Conflict on interest 

MP, MA, LL, CHP, YL, and SZ: Employees of Celgene Corporation. AW: Employee of Novartis Institutes for BioMedical Research. IS: Employee of Bristol-Myers Squibb. 

**Table 1.** Summary of apremilast, M12, and M14 plasma pharmacokinetic parameters on day 5 following multiple oral doses of apremilast (geometric mean, geometric CV%).

	Apremilast 30 mg b.i.d.			Apremilast 50 mg b.i.d.		
	Apremilast n = 53	M12 n = 53	M14​ n = 53	Apremilast n = 55	M12 n = 55	M14 n = 55
t_max_^a^ (h)	2.0 (0.5 – 6.0)	3.0 (1.0 – 12.0)	3.0 (0.5 – 12.0)	2.0 (0.6 – 6.0)	3.0 (0.5 – 6.0)	3.0 (0.5 – 6.0)
C_max_ (ng/mL)	351.8 (37.1)	44.2 (27.7)	41.8 (41.1)	507.2 (32.3)	659.1 (21.0)	62.2 (35.3)
AUC_0–_τ (ng×h/mL)	2,260 (36.1)	3,930 (23.9)	363.8 (35.9)	3,451 (38.6)	5,968 (20.0)	544.9 (33.9)
t_1/2_ (h)	6.41 (24.3)^b^	12.18 (25.9)^c^	19.20 (49.0)^d^	7.28 (29.1)	12.79 (29.0)^e^	20.31 (57.1)^f^

^a^The t_max_ is summarized by median and range (minimum–maximum); ^b^n = 52 for half-life calculations; ^c^n = 35 for half-life calculations; ^d^n = 10 for half-life calculations; ^e^n = 46 for half-life calculations; ^f^n = 19 for half-life calculations. b.i.d. = twice daily; CV% = coefficient of variation; C_max_ = maximum plasma concentration; t_max_ = time to maximum plasma concentration; t_1/2_ = estimate of the terminal elimination half-life in plasma; AUC_0–_τ = area under the plasma concentration-time curve from time 0 to the dosing interval τ (the dosing interval is 12 hours).
